# Zoledronate Triggers Vδ2 T Cells to Destroy and Kill Spheroids of Colon Carcinoma: Quantitative Image Analysis of Three-Dimensional Cultures

**DOI:** 10.3389/fimmu.2018.00998

**Published:** 2018-05-08

**Authors:** Serena Varesano, Maria Raffaella Zocchi, Alessandro Poggi

**Affiliations:** ^1^Molecular Oncology and Angiogenesis Unit, Ospedale Policlinico San Martino, Genoa, Italy; ^2^Division of Immunology, Transplants and Infectious Diseases, San Raffaele Scientific Institute, Milan, Italy

**Keywords:** CRC, spheroid, γδ T cells, epidermal growth factor receptor, antibody-dependent cellular cytotoxicity, zoledronate

## Abstract

New successful anti-cancer strategies are based on the stimulation of immune reaction against tumors: however, preclinical testing of such treatments is still a challenge. To improve the screening of anti-cancer drugs, three-dimensional (3D) culture systems, including spheroids, have been validated as preclinical models. We propose the spheroid 3D system to test anti-tumor drug-induced immune responses. We show that colorectal carcinoma (CRC) spheroids, generated with the epithelial growth factor (EGF), can be co-cultured with Vδ2 T cells to evaluate the anti-tumor activity of these effector lymphocytes. By computerized image analysis, the precise and unbiased measure of perimeters and areas of tumor spheroids is achievable, beside the calculation of their volume. CRC spheroid size is related to ATP content and cell number, as parameters for cell metabolism and proliferation; in turn, crystal violet staining can check the viability of cells inside the spheroids to detect tumor killing by Vδ2 T cells. In this 3D cultures, we tested (a) zoledronate that is known to activate Vδ2 T cells and (b) the therapeutic anti-EGF receptor humanized antibody cetuximab that can elicit the antibody-dependent cytotoxicity of tumor cells by effector lymphocytes. Zoledronate triggers Vδ2 T cells to kill and degrade CRC spheroids; we detected the T-cell receptor dependency of zoledronate effect, conceivably due to the recognition of phosphoantigens produced as a drug effect on target cell metabolism. In addition, cetuximab triggered Vδ2 T lymphocytes to exert the antibody-dependent cellular cytotoxicity of CRC spheroids. Finally, the system reveals differences in the sensitivity of CRC cell lines to the action of Vδ2 T lymphocytes and in the efficiency of anti-tumor effectors from distinct donors. A limitation of this model is the absence of cells, including fibroblasts, that compose tumor microenvironment and influence drug response. Nevertheless, the system can be improved by setting mixed spheroids, made of stromal and cancer cells. We conclude that this type of spheroid 3D culture is a feasible and reliable system to evaluate and measure anti-tumor drug-induced immune responses beside direct anti-cancer drug effect.

## Introduction

It is known that the immune system can control both survival and proliferation of tumors (1–3). Among anti-tumor lymphocytes, γδ T cells can be triggered by multiple stimuli elicited by transforming cells; for this reason they represent a category of effector cells employable in immunotherapy (4, 5). T lymphocytes of the Vδ2 subset that express the Vγ9 chain (Vγ9Vδ2) represent the majority of γδ T cells (6). At variance with αβ T cells, Vγ9Vδ2 T lymphocytes recognize non-peptide phosphorylated small molecules, namely phosphoantigens (PAg), that allow their *in vivo* and *in vitro* activation and expansion (7–10). In mammalian cells, a physiologic PAg recognized by Vγ9Vδ2 T lymphocytes is the isopentenylpyrophosphate (IPP), one of the mevalonate pathway products (8–10). The ability of IPP to trigger Vγ9Vδ2 T lymphocytes is thought to be mediated by the recognition *via* T cell receptor (TCR) (11–13). Pharmacological treatment with amino bisphosphonates (N-BPs), such as zoledronate (Zol), blocking the farnesyl pyrophosphate synthase of the mevalonate pathway, leads to IPP accumulation in tumor cells and, as a consequence, to the activation and expansion of Vγ9Vδ2 T lymphocytes (11–13). As final outcome, the expanded γδ T cell population is able to produce anti-cancer cytokines and exert anti-tumor cytotoxicity. N-BPs have been used in the treatment of many kind of bone diseases, including osteoporosis and bone tumors (14). Besides, due to their role as potent activators of γδ T lymphocytes, these compounds have been proposed for cancer immunotherapy (5, 14, 15). In addition, γδ T cells expressing the FcγRIIIA (CD16) can kill tumor cells by antibody-dependent cellular cytotoxicity (ADCC); this mechanism can be exploited using therapeutic antibodies (16).

On the basis of the promising potential of γδ T lymphocytes as anti-cancer agents *in vitro*, several clinical trials have been launched in the last 10 years for patients with hematological and non-hematological malignancies (5). Despite several encouraging results in multiple myeloma and non-Hodgkin lymphoma, γδ T cell-based immunotherapy gave disappointing results in different solid tumors, raising the question of whether tissue characteristics and/or microenvironment deeply contribute to determine the outcome of treatments (17). This indicates that preclinical models that allow the definition of drug safety and efficacy are needed. However, to test immunosurveillance in animal models is difficult; to this purpose humanized mice, EBV-infected animals with activated T cells, or the NOG, NSG engrafted with CD34^+^ cells or peripheral blood lymphocytes and patient-derived tumor xenografts have been used (18–20). This approach, mostly used in studies on the pharmacological control of immunocheckpoints, is very expensive and requires a long time to be set up and defined for each tumor. Also, there is increasing evidence that tumor development in humans is not always reproducible and predictable in other animals, in particular when extremely artificial models are used (21–24). As an alternative, several three-dimensional (3D) culture systems, including spheroids, have been validated by the European Union Reference Laboratories for Alternatives to Animal Testing (EURL ECVAM) as preclinical models, to overcome these inconveniences (25–28).

In this context, we propose the spheroid 3D culture system to evaluate anti-tumor drug-induced immune response; in particular, we analyzed the anti-tumor effects of Vδ2 T lymphocytes triggered by Zol and/or the anti-epidermal growth factor receptor (EGFR) humanized antibody (huAb) cetuximab (Cet) on different CRC cell lines. We show that 1. Spheroids can be a reliable 3D culture system that allows co-culture of tumor cells and effector lymphocytes. 2. Zol and/or Cet triggers Vδ2 T cells to kill and degrade spheroids of colorectal carcinoma (CRC) cells. 3. The effects of drugs and effector cells can be measured by computerized imaging.

## Materials and Methods

### Cell Cultures

The human CRC cell lines Caco2, HT29, HCT15, SW480, DLD1, HCT116, LS180, WiDr, LoVo, Colo205, Colo320 DMF, SW620, T84, and SW48, were from the cell bank of the Policlinico San Martino (kind gift of Blood Transfusion Centre, Dr. Barbara Parodi). CRC cell lines in adherent cultures were maintained in RPMI-1640 (Gibco, Life Technologies Italy, Monza) medium supplemented with 10% fetal serum (FBS, Gibco™ One Shot™ Fetal Bovine Serum, Thermo Fisher Scientific Italy, Monza, Italy), penicillin/streptomycin and l-glutamine (BioWhittaker^®^ Reagents, Lonza, Basel, Switzerland) in a humidified incubator at 37°C with 5% CO_2_.

### Immunofluorescence Assay and Cytofluorimetric Analysis

Immunofluorescence assay was performed as described (29) with the anti-ICAM1 monoclonal antibody (mAb) (14D12D2, IgG1) (29), or anti-CD133-specific mAb (W6B3C1, IgG1, Miltenyi Biotec, Bergish Gladbach, Germany) on CRC cell lines or with anti-Vδ2 mAb (BB3, IgG1) (30) or anti-CD3 mAb (289/10/F11, IgG2a) or anti-CD16 mAb (VD4, IgG1) (29) on Zol-stimulated lymphocytes, followed by Alexafluor647 anti-IgG2a or PE-anti-IgG1 goat anti-mouse antiserum (GAM) (Life Technologies, Milan, Italy). The Fc chimeras (soluble receptors fused with the Fc of human immunoglobulins): Fc-NKG2D and Fc-DNAM1 were purchased from R&D System (Minneapolis, MN, USA) and used on CRC cell lines in immunofluorescence assay followed by Alexafluor647 goat anti-human antiserum (Life Technologies). At least 5,000 cells/sample were run on a CyAn ADP cytofluorimeter (Beckman-Coulter Italia, Milan, Italy) and results analyzed with the Summit 4.3 software.

### Tumor Spheroid Generation

Optimal experimental conditions for the generation of tumor cell spheroids were selected starting from decreasing number of each tumor cell line (2 × 10^4^–1 × 10^4^–5 × 10^3^ per well) in flat-bottom 96-well plates (Ultra-Low attachment multiwell plates, Corning^®^Costar^®^, NY, USA) with DMEM-F12 (BioWhittaker^®^ Reagents, Lonza) in serum free medium (SFM), supplemented with epithelial growth factor (EGF) (Peprotech Europe, London, UK) at 10 ng/ml final concentration (≥1 × 10^6^ U/mg). EGF was selected, among three other natural ligands of EGFR [transforming growth factor-α (TGFα) (≥5 × 10^6^ U/mg); amphiregulin (AREG), ED_50_ for proliferative effect is 5–10 ng/m; and epiregulin (EPN), ≥5 × 10^5^U/mg; all from Peprotech], to obtain proliferation of CRC cells as spheroids, compared to cell growth determined in two-dimensional (2D) conventional cell culture conditions, evaluated by measurement of ATP content along time. Generation of spheroids was monitored till day 14 and proliferation and dimension (perimeter, area, and volume) were analyzed in each culture well. At least triplicates were analyzed for each culture condition and the number of spheroids analyzed is indicated in each figure (a minimum of 150 single spheroids for each independent experiment). Experiments were performed on day 6 of spheroids formation (Figures S1A,B in Supplementary Material). This day was chosen as at this time all cells in culture were alive and the diameter of spheroids was of about 250 µm.

### *Ex Vivo* Expansion of Vδ2 T Cells

Peripheral blood mononuclear cells (PBMC) were obtained from healthy adult donor’s buffy coat (institutional informed consent signed at the time of donation and EC approval PR163REG2014) by density gradient centrifugation using Lymphocyte Separating Medium (Pancoll human, Density: 1.077 g/ml, PAN-Biotech, Munich, Germany) as described (29). To obtain Vδ2 T lymphocyte populations, 10^5^ PBMC were cultured in 96 W U-bottomed plates in 200 µl of RPMI-1640 (Gibco Life Technologies) medium supplemented with 10% FBS (Gibco™), penicillin/streptomycin and l-glutamine (BioWhittaker^®^ Reagents) and with 1.0 µM zoledronic acid as zoledronate (Zol, Selleckem, Munich, Germany) at 37°C humidified cell incubator with 5% of CO_2_. After 24 h, and on day 5 and 7, 100 µl of culture medium were discarded and 100 µl of recombinant human IL-2 (30 IU/10 ng/ml final concentration, Miltenyi Biotec Italia, Bologna) were added to the cultures (Figures S1A,B in Supplementary Material). The percentage of Vδ2 T lymphocyte was determined at different time points (day 0, 7, 10, 14, 21) by indirect immunofluorescence and cytofluorimetric analysis using the anti-Vδ2 TCR-specific mAb BB3 (30) followed by isotype-specific Alexafluor647 conjugated goat anti-mouse antiserum (GAM, Life Technologies) as described (Figure S1C in Supplementary Material, left plot) (29). Lymphocyte populations were used as effector cells in co-cultures experiment with tumor cell spheroids after day 21, when the percentage of Vδ2 lymphocytes were more than 96% of total cells (Figure S1C in Supplementary Material, central graph). At that time, the majority of cells expressed the FcγRIII CD16 (Figure S1C in Supplementary Material, right histogram). In some experiments, Vδ2 lymphocytes were separated from PBMC using the positive Easy-Sep Do-It-Yourself Selection Kit (Stemcell Technologies, Vancouver, BC, Canada): the purity of separation was always more than 96% and the recovery about 75%. Vδ2 T lymphocytes were cultured in 200 µl in U-bottomed plates with 10 µg/ml of phytoemoagglutinin A (PHA, Sigma Chemical Co., St. Louis, MO, USA) and 30 IU/ml (10 ng/ml) of IL-2 (Miltenyi Biotech).

### CRC Spheroids and Vδ2 T Cell Co-Cultures

On day 6, spheroids with a maximal diameter of 250 µm were composed of living cells, as assessed by culturing a sample under adherent conventional conditions for 12 h and subsequent identification of living cells with propidium iodide (PI, Sigma) staining, ATP content, and crystal violet assay. In preliminary experiments, the number of CRC cells present in a culture well containing spheroids was determined measuring the ATP content referred to the ATP content of the same cell line at a known cell concentration in conventional culture system. The number of cells present in a given tumor spheroid was determined calculating the ratio between spheroid volume and the volume of a single cell, measuring the diameter of a cell and assuming that this cell displayed a spherical shape. Co-cultures were set up by adding decreasing amount of Vδ2 T cells (1.5 × 10^5^, 0.75 × 10^5^, and 0.35 × 10^5^) to spheroids of CRC cells on day 6 and further culture in RPMI SFM for different periods of time (4, 12, 24, and 48 h). The optimal amount of Vδ2 T cells to detect the cytotoxic effect, determined in preliminary experiments by crystal violet assay or by image analysis, was 0.75 × 10^5^ cells/well and it corresponded approximately to a 1:1 effector to target (E:T) ratio. The time point selected was 24 h based on CRC spheroid damage evaluated by microscopy and image analysis. In some samples, the anti-Vδ2 mAb BB3 (5 µg/ml) was added. In other experiments, the anti-EGFR therapeutic antibody cetuximab (Cet, obtained as Erbitux left over from the Antiblastic Unit of Policlinico San Martino) was added at the concentration of 2 µg/ml to evaluate the ADCC exerted by Vδ2 T cells. CRC cell lines used in these experiments were selected on the basis of expression of EGFR (reactivity with Cet) and the ability of forming spheroids.

### PI Staining, ATP Content, and Crystal Violet Assay

To determine cell membrane permeability, cells were stained with a PI solution (50 µg/ml) and incubated 15 min at RT. After extensive washing, at least 5,000 cells/sample were run on a CyAn ADP cytofluorimeter (Beckman-Coulter) and results analyzed with the Summit 4.3 software. ATP content was determined using the CellTiter-Glo^®^ Luminescent Cell Viability Kit (Promega Italia Srl, Milan, Italy) following manufacturer’s instruction using the luciferase reaction consisting in mono-oxygenation of luciferin catalyzed by luciferase in the presence of Mg^2+^, ATP, and molecular oxigen. Luminescence was detected with the VICTORX5 multilabel plate reader (Perkin Elmer, Milan, Italy) expressed as relative light units (RLU); in some instances the, RLU were converted in µM of ATP according to a standard curve. The crystal violet assay was performed with the Crystal Violet Cell Cytotoxicity Assay Kit (Biovision, Milpitas, CA, USA). Briefly, CRC spheroids alone or co-cultured with Vδ2 T cells in the presence or not of Zol were transferred in conventional adherent plates and after 48 h were stained with crystal violet following manufacturer’s instruction. After extensive washing, adherent cells were solubilized and the amount of crystal violet proportional to the amount of living cells was measured with the VICTORX5 multilabel plate reader (Perkin Elmer) at the optical density (OD) of 595 nm.

### Images and Measurement of Spheroid Size

Cell cultures were analyzed with Olympus IX70 bright field inverted microscope equipped with a CCD camera (ORCA-ER, C4742-80-12AG, Hamamatsu, Japan), associated with the CellSens software (version 1.12, Olympus, Tokyo, Japan). Images were taken with 10× objective NA 0.30 or 20× objective NA 0.40 (100× or 200× magnification). After calibration of the plate, a grid corresponding to each well was created and analyzed; at least nine focal points were taken for each well of interest to obtain optimal images to be measured. After this operation through an automated *x*–*y* axis motorized table SCAN IM 120 × 100-2 mm (Marzhauser Wetzlar GmBH, 35579 Wetzlar, Germany) images of the entire well were taken. The dimension of the camera chip was 2/3-inch format, thus images were partly overlapped (from 15 to 35%) by the software to obtain appropriate stitched images (usually 50 images for each well were taken and 3–6 replicates performed for each experimental condition). The final result was an image at 16-bit gray scale with a size of 1,102 × 9,626 pixel (7.1 mm × 6.2 mm) in .csi format that can be analyzed with the CellSens software. The measurement of spheroids was performed manually by applying the “Count and Measure” tool of the CellSens software. This tool allows to get circular region of interest (ROI) that can represent the perimeter of the spheroid. Through calibration of the microscope, the number of pixel of the image were converted into μm, the diameter of each spheroid was calculated and the data were inserted in an Excel sheet with a defined color corresponding to a ROI and to a spheroid. Data were then transferred to GraphPad Prism computer program (version 5.03) for subsequent statistical analysis and generation of graphs (see Results). To avoid counting and measuring very small spheroids or single cells or small cell/debris aggregates, only spheroids with a diameter greater than 50 µm were selected. The perimeter of spheroids was identified by the different image contrast with the bottom of the well. Spheroids that did not show an approximately spherical shape, but appeared as the fusion/overlap of several spheroids, were considered as composed of different single spheroids depicting partly overlapping ROI.

### Statistical Analysis

Data are presented as mean ± SEM or ±SD. Statistical analysis was performed using two-tailed unpaired Student’s *t* test. The cutoff value of significance is indicated in each figure legend.

## Results

### Generation and Measurement of CRC Spheroids

**Figure 1:**
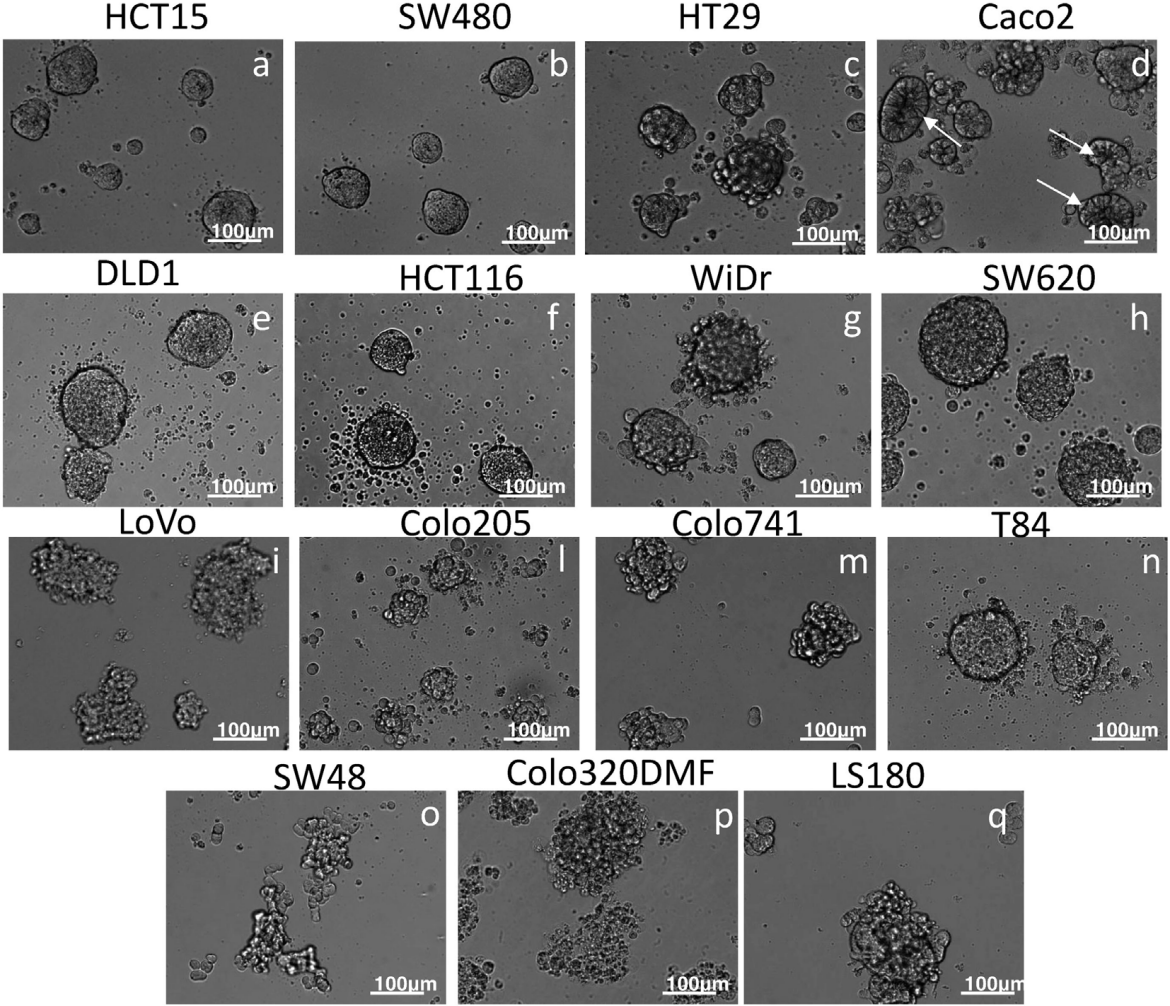
Generation of CRC spheroids. The indicated CRC cell lines were cultured in serum free medium supplemented with 10 ng/ml of epithelial growth factor in very low adherent 96-well flat-bottomed microplates and analyzed on day 6 by inverted IX70 microscope (Olympus); images were taken with 20× objective NA 0.40 (200× magnification). Bar in each panel: 100 µm. Some spheroids were characterized by a spherical-like appearance with smooth surface **(a,b,e,f,h,n)**, others displayed rough surface **(c,d,g,i–m)**. In some instances (HT29, Colo205, Colo741, and T84), on the outer surface or next to the spheroid single cells are present. SW48, Colo320DMF, and LS180 **(o–q)** formed unshaped cell aggregates. In the case of Caco2, cell line the central portion of the spheroid appeared darker (arrows) than the peripheral region, suggesting a crypt-like morphology.

First, we determined which CRC cell lines can grow and give rise to spheroids under controlled culture conditions. To this aim, CRC cells were cultured in SFM, without or with EGF (10 ng/ml), in ultra low adherent plates and analyzed microscopically from day 1 to 7 of culture. We found that not all the CRC cell lines tested give rise to spheroids under these experimental conditions. Indeed, HCT15, SW480, HT29, Caco2, DLD1, HCT116, WiDr, SW620, LoVo, Colo205, Colo741, and T84 (Figures 1a–n) could form spheroids, while SW48, Colo320DMF, and LS180 cell lines simply led to cell aggregates where single cells were still distinguishable on day 7 of culture (Figures 1o–q). HCT15, SW480, DLD1, HCT116, SW620, and T84 spheroids displayed a smooth surface (Figures 1a,b,e,f,h,n), while other cell lines, including HT29, Caco2, LoVo, Colo205, and Colo741, spheroids showed a rough surface (Figures 1c,d,i,l,m) or smooth and rough surface such as WiDr (Figure 1g). It is of note that spheroids obtained with Caco2 cells appeared with a central portion darker than the periphery, suggesting that the culture conditions used can generate crypts-like structures (Figure 1d). The ability to form spheroids was not related to the expression of CD133 marker (Figure S2A in Supplementary Material); indeed, some cell lines able to form spheroids were completely negative for this marker (HCT15, SW480, DLD1, Colo205, Colo741, and T84) while SW48 cell line was CD133^+^ but did not give rise to spheroids.

The expression on tumor cells of surface molecules involved in the interaction with effector lymphocytes was comparable to that displayed by the same cell line in conventional cultures in adherent plates. As an example, the expression of ICAM1, the ligand for LFA1, was similar in SW480 cells from 2D cultures or disaggregated spheroids (Figure S2B in Supplementary Material for SW480, not shown HCT15, HT29, and WiDr). Likewise the chimeric Fc-NKG2D and Fc-DNAM1 activating receptors could equally bind CRC cells derived from spheroids or conventional 2D cultures (Figure S2B in Supplementary Material for SW480; not shown HCT15, HT29, and WiDr).

**Figure 2:**
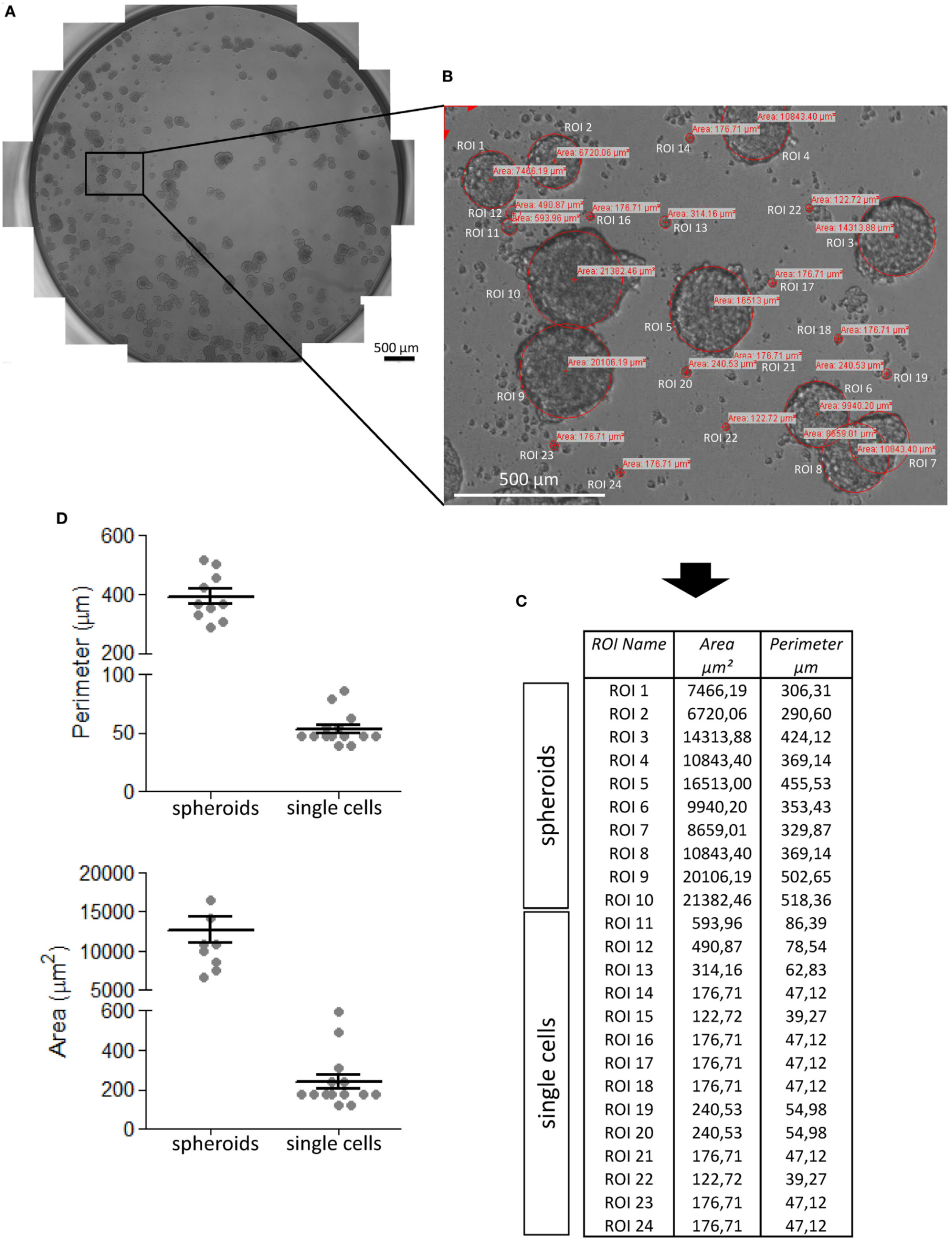
Procedure of CRC spheroid image analysis and measurement. CRC spheroids (this example is referred to SW620 cell line) were analyzed under an inverted IX70 microscope (Olympus) with 10× objective NA 0.30 (100× magnification), equipped with an automated *x*–*y* axis motorized table SCAN IM (Marzhauser Wetzlar) and image taken under control of the CellSens computer program. **(A)** The entire well was reconstructed taking sequential and partially overlapping images, stitched one to each other to give rise to the image of the well. **(B)** Region of interest (ROI) that identify tumor cell spheroids and areas (μm^2^) calculated by CellSens software after calibration of the image. **(C)** Data inserted by the CellSens software in an Excel sheet were analyzed with Graph Pad PRISM (version 5.03) software **(D)**. **(D)** Perimeters (upper graph) and areas (lower graph) of spheroids and single cells. Mean ± SEM of the ROI shown in panel **(B)** and measured in panel **(C)**.

In order to determine the size of spheroids present in a culture well, we applied a manual method using a microscope equipped with a motorized *x*–*y* table and a CCD camera (see Materials and Methods). As shown in Figure 2, to determine spheroid size, the whole area of a given culture well was reconstructed by CellSens software using single images then partly overlapped (Figure 2A, final 100× magnification of the image). The *z*-focus was pre-determined manually, focusing random from 3 to 5 individual points for each well, to avoid interferences due to the irregular surface of culture wells. Each well image was subjected to computerized analysis of single spheroids and their measurement. The ROI were manually depicted with the CellSens software tool that uses three points to get a circle, assuming that the 3D spheroids displayed a circular perimeter when observed in 2D (Figure 2B). The perimeter and area of each spheroid could be determined by a measure tool of the software; Figures 2C,D show these measures for spheroids and single cells.

**Figure 3:**
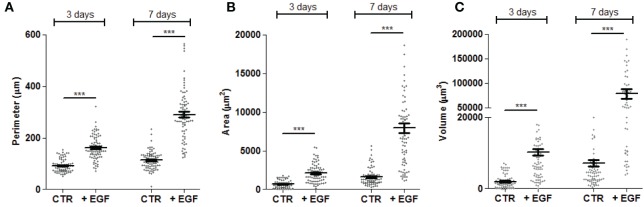
Measurements of perimeter, area, and volume of CRC spheroids. The perimeter **(A)**, area **(B)**, and volume **(C)** of each spheroid was calculated as explained in Figure 2 and data were plotted with Graph Pad PRISM software. This example is referred to the HCT15 cell line cultured as indicated in Figure 1. Spheroid dimensions were evaluated after 3 and 7 days of culture without epithelial growth factor (EGF) (CTR) or with 10 ng/ml EGF. Each symbol indicates a region of interest which in turn corresponds to a single spheroid. Bar: mean ± SEM of that group of measures. ***p < 0.001.

The evaluation of these parameters in one representative experiment using the CRC cell line HCT15 is shown in Figure 3. Addition of EGF to cell cultures triggered a strong increment in the size of spheroids and this effect was already detectable and statistically significant on day 3. The growth of spheroids was much more evident at day 7 and at this time point spheroids were of heterogeneous size, ranging from a perimeter of 150 µm to more than 500 µm (Figure 3A). Likewise, area and volume (Figures 3B,C) were significantly increased by day 3 and more evident on day 7. It is of note that it was possible to calculate mathematically the number of cells in a given tumor spheroid, assuming that the spheroid volume is the sum of volumes of the single cells (data not shown). This allowed to calculate that the number of cells for each spheroids was less than 500 to more than 3,000 (data not shown). To define whether this analysis was objective and reproducible, avoiding biases due to the operator, results of independent measurement have been compared. As shown in Figure S3 in Supplementary Material, no significant differences were found in the evaluation of SW620 or HCT15 spheroid perimeters (Figure S3A in Supplementary Material) and areas (Figure S3B in Supplementary Material) by three operators.

**Figure 4:**
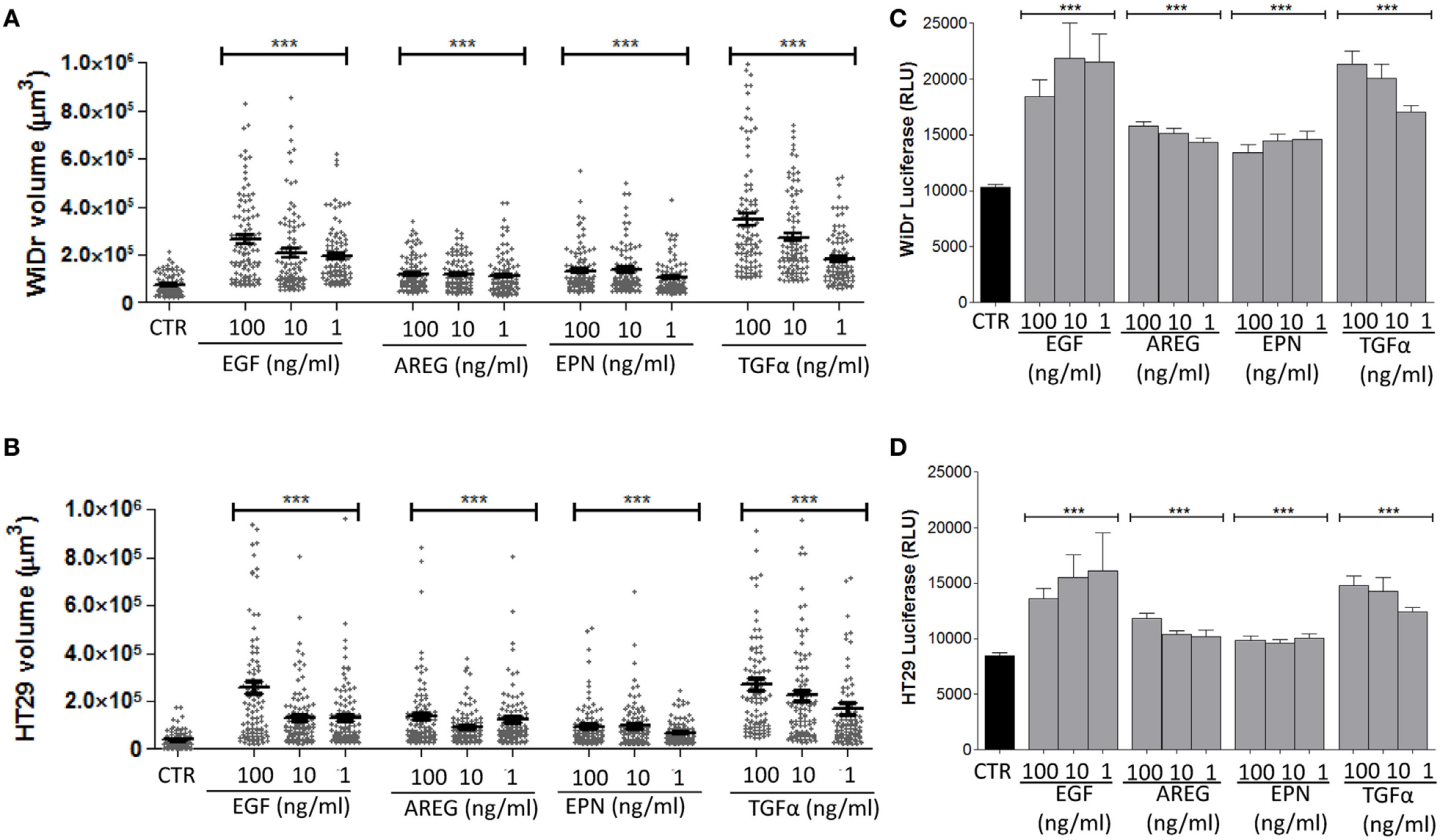
Effect on spheroid size of different epidermal growth factor receptor ligands. **(A,B)** CRC cell lines [WiDr **(A)**; HT29 **(B)**] were cultured in serum free medium (SFM) without (CTR) or with 100-10-1 ng/ml of epithelial growth factor (EGF), amphiregulin (AREG), epiregulin (EPN), or transforming growth factor-α (TGFα), in very low adherent 96-well flat-bottomed microplates. Cell cultures were analyzed on day 6 by inverted IX70 microscope (Olympus) and images were taken with 10× objective NA 0.30 (100× magnification). Each symbol in the different plots indicates a spheroid. Data are shown as volume (μm^3^). Bar: mean ± SEM of that group of measures. ***p < 0.0001. **(C,D)** ATP content of the same cultures, expressed as luminescence of luciferase activity. Black columns indicate the ATP content of cells cultured in SFM and gray columns the ATP content of cell cultured in the presence of the indicated factors. Data are the mean ± SD of 6-well replicates for each culture condition. ***p < 0.001.

In another series of experiments, we assessed whether the evaluation of spheroids size can allow to identify differences among the efficiency in spheroid generation of EGFR ligands other than EGF, including AREG, EPN, and TGFα. We observed that all these ligands could lead to spheroids of WiDr (Figure 4A), HT29 (Figure 4B), SW620, and Caco2 (data not shown) cell lines. Indeed, a significant increase of spheroid volume was detectable with all the EGFR ligands used compared to spheroids size in SFM not supplemented with these factors (Figures 4A,B). The fold of size increment was related to the amount of the EGFR ligand used. For instance, with TGFα the volume of spheroids of WiDr cell line was sixfold, fivefold, and threefold larger at 100, 10, and 1 ng/ml than that of spheroids in medium without TGFα (Figure 4A). Furthermore, a stronger effect on the increase of spheroids volume was found with EGF and TGFα, compared to AREG and EPN (Figures 4A,B, *p* < 0.001). Importantly, an increase in ATP cell content corresponded to the increase of spheroids size (Figures 4C,D) supporting the hypothesis that spheroid volume is related to an increment of cell metabolism and proliferation. Indeed, experiments with HCT15, HT29, Caco2, and SW480 CRC cell lines showed a direct relationship between ATP content and cell number (Figure S4A in Supplementary Material).

### Analysis of the Effect of Zol and Vδ2 T Lymphocytes on CRC Spheroids

Tumor spheroids are currently used to assess the effect of cytotoxic drugs as models for *in vitro* therapeutic screening (25–28). Indeed, 3D tumor cell cultures show several *in vivo* features of tumors, including drug response and resistance (25–28). To our knowledge, the analysis of effector lymphocyte activity on tumor cell spheroids has not been performed yet. Some reports have analyzed the effect of anti-cancer lymphocytes on the killing of CRC cells isolated from tumor spheroids (31). This means that spheroids were first disaggregated and a single cell suspension was then used in conventional cytolytic experiments. In our hands, a single tumor cell suspension could be obtained by gently harvesting and transferring spheroids in phosphate buffered saline or culture medium. However, we observed that in some CRC cell lines, the percentage of tumor cells stained with PI was higher among cells derived from disaggregated spheroids than from adherent cultures (Figure S4B in Supplementary Material, central vs left subpanels). This strongly suggests that cell suspensions obtained from spheroids may have an altered membrane permeability. Conversely, intact CRC spheroids transferred into adherent plates showed a percentage of PI^+^ cells comparable to that of CRC cells cultured under conventional conditions (Figure S4B in Supplementary Material, right vs left panels). Thus, to evaluate the effect of Zol or Vδ2 T cells, preformed spheroids of CRC cell lines were used. In any case, in each experiment we assessed the vitality of CRC spheroids, besides evaluating their size. To this aim, in parallel with image analysis, measurement of ATP content and staining with crystal violet were performed. Both these tests provide information about the vitality of cells in a spheroid: indeed, ATP content is directly related to cell metabolism and proliferation, while crystal violet works as an intercalating compound in DNA, thus staining the nuclei and allowing the quantitative determination of living cells that are able to adhere to the culture plate.

**Figure 5:**
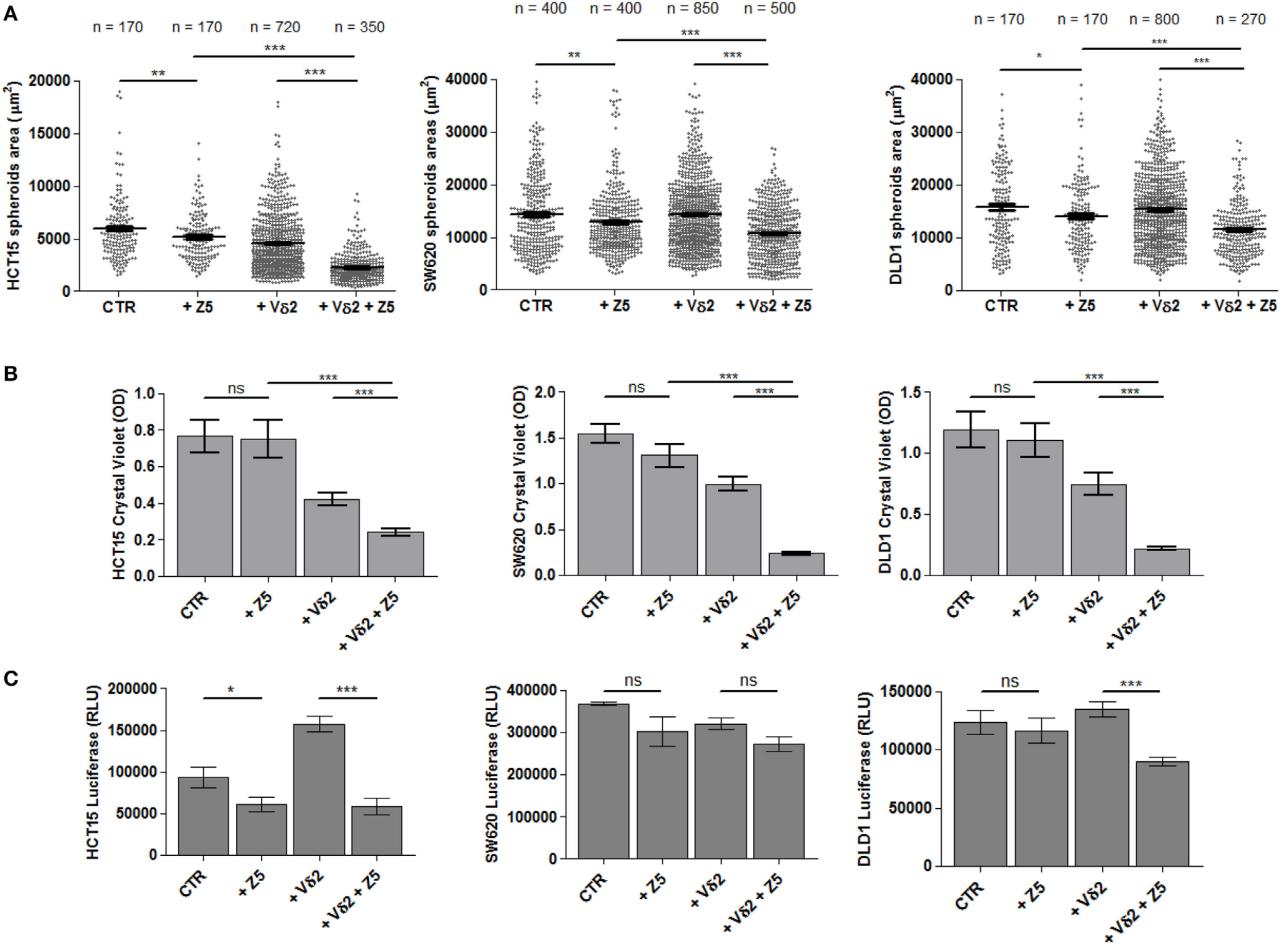
Effect of Zol and Vδ2 T cell populations on CRC spheroid size and viability. **(A)** Spheroids from different CRC cell lines (HCT15, left; SW620, middle; and DLD1, right) were generated in serum free medium supplemented with epithelial growth factor (10 ng/ml). On day 6, culture wells were incubated for additional 24 h without (CTR) or with 5 µM Zol (+Z5) or Vδ2 T cells (+Vδ2) or Vδ2 T cells + 5 µM Zol (+Vδ2+Z5). Then, each culture well was analyzed for the measurement of spheroids. The number of spheroids analyzed is indicated in the upper part of each plot; data are from experiments performed with six different Vδ2 T cell populations in triplicate wells for each donor. Each symbol in the plot indicates a single spheroid. Bars: mean ± SEM of that group of measures. *p < 0.01; **p < 0.001; ***p < 0.0001. **(B)** Cells from the different cultures defined in panel **(A)**, were gently harvested and transferred to conventional 96-well flat-bottomed plates to allow the adhesion of CRC epithelial cells. After 48 h, cell cultures were labeled with crystal violet followed by extensive washes (to remove non-adherent cells) and lysis of adherent cells. Optical density (OD) at 595 nm was evaluated with a VICTORX5 analyzer. Bars: mean ± SD of three experiments. ***p < 0.0001; ns, not significant. **(C)** Cell cultures obtained as indicated in panel **(A)** were analyzed for ATP content with luciferase assay. Data are shown as luminescence in relative light units (RLU) arbitrary units determined with a VICTORX5 analyzer. Bars: mean ± SD of three experiments.

The spheroids size of the three representative CRC cell lines HCT15, SW620, and DLD1 was analyzed upon incubation for 24 h with either Zol (5 µM), or Vδ2 T lymphocytes or with Zol and Vδ2 T cells. Data from independent experiments, performed with different bulk Vδ2 T cell populations from six healthy donors, have been pooled together providing a very large number of spheroids evaluated for each cell line analyzed. Figure 5A shows that the combination of Vδ2 T cells and Zol led to a strong decrease of spheroid size in all CRC cell lines. This effect was striking with HCT15 cell line, where the size of tumor spheroids was reduced to one/third (Figure 5A, left graph). Zol alone can slightly reduce the size of HCT15, SW620 and DLD1 cell lines, while the reductive effect due to Vδ2 T cells, in the absence of Zol, was evident only using HCT15 as target cells (Figure 5A, left graph). When the Vδ2 T cell populations obtained from the six different donors were analyzed independently (Figure S5 in Supplementary Material), we found that Zol could trigger anti-tumor Vδ2 T lymphocytes in four donors (donors 1–4), while in one donor Vδ2 T cells were efficient also in the absence of Zol (donor 5), and in one case (donor 6) were not effective even in the presence of Zol.

The parallel evaluation of crystal violet assay (Figure 5B) and measurement of ATP content (Figure 5C) complete the analysis of the effect of Zol and/or Vδ2 T cells on tumor cell spheroids. Crystal violet assay was performed after gently harvesting spheroids and subsequent culture in conventional adherent plates for 48 h; this allowed living tumor cells to adhere to plastic before staining. Non-adherent cells (dying tumor cells and Vδ2 T cells present in culture) were discarded upon extensive washing, and plates were read on a spectrophotometer. As shown in Figure 5B, a strong reduction of OD in cultures of HCT15, SW620, and DLD1 spheroids with Vδ2 T cells was detected, indicating that most CRC cells have been killed; this effect was significantly enhanced by Zol.

ATP content was measured at 24 h (at the same time point of the evaluation of spheroids size): the ATP detected in the cultures with Zol (5 µM) and Vδ2 T cells (Figure 5C, +Vδ2+Z5) was lower than that of either CRC spheroids (CTR) or co-cultures of Vδ2 T cells and CRC spheroids (+Vδ2). Zol alone determined a slight decrease of ATP when added to cell spheroids of any CRC cell line tested. These data suggest a reduced metabolism and/or proliferation in cultures with low ATP content, conceivably due to Vδ2 T anti-CRC activity. However, the addition of Vδ2 T cells to HCT15 spheroids led to an increment of ATP content (Figure 5C, left graph), making difficult to discriminate the ATP of effector lymphocytes and of tumor cells.

### CRC Spheroids to Evaluate TCR Involvement in Vδ2 T Cell Killing and ADCC Triggered With Cetuximab

**Figure 6:**
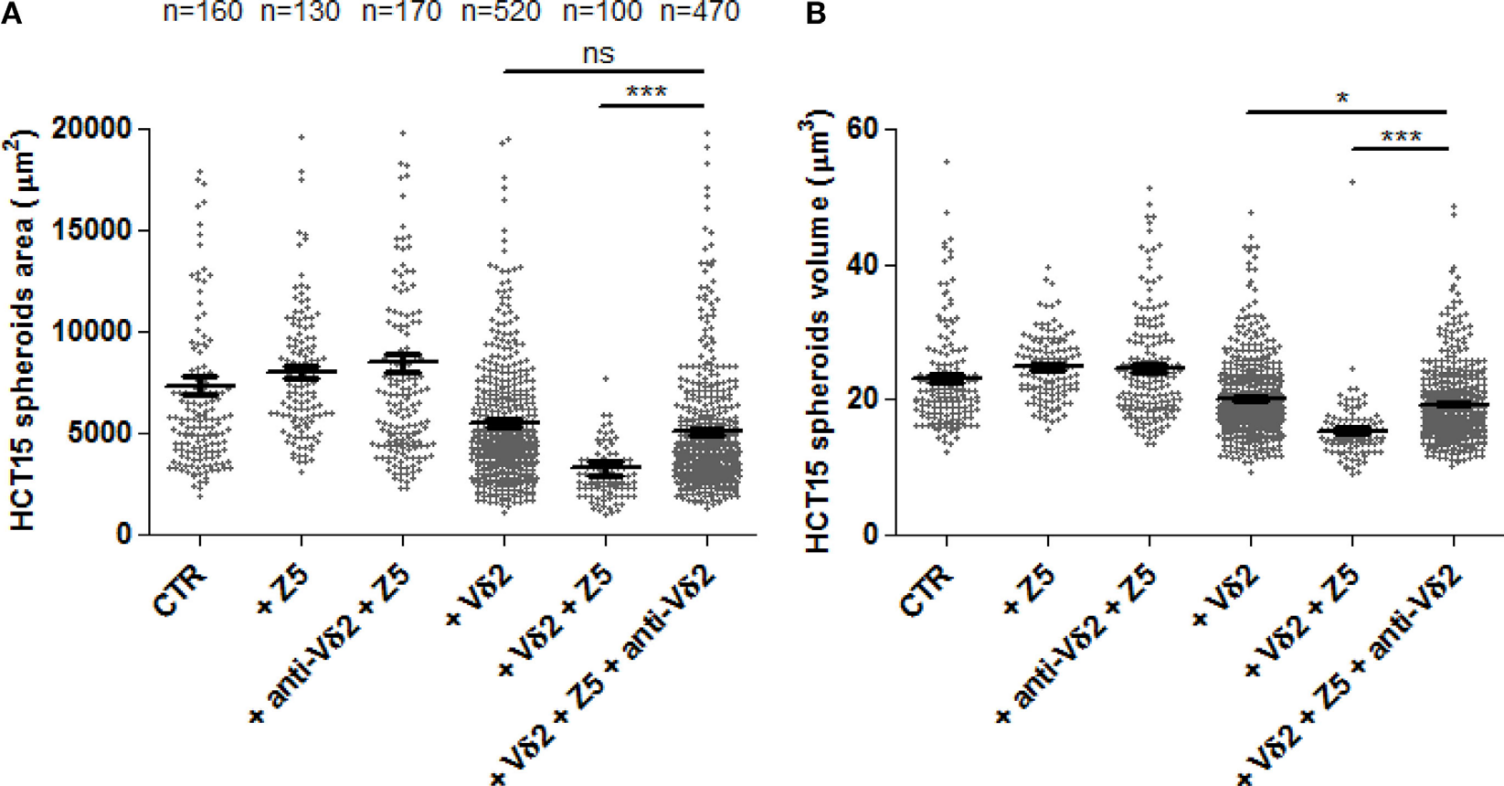
The effects of Zol and Vδ2 T cells on CRC spheroids are dependent on Vδ2TCR. HCT15 spheroids were generated in serum free medium supplemented with epithelial growth factor (10 ng/ml) and incubated on day 6 for additional 24 h in medium without (CTR) or with 5 µM Zol (+Z5) or Vδ2 T cells (+Vδ2) or Vδ2 T cell populations + 5μM Zol (+Vδ2+Z5). In some experiments, saturating amounts of the anti-Vδ2-specific monoclonal antibody BB3 (5 µg/ml) were added at the onset of the 24 h incubation (+anti-Vδ2+Z5, +Vδ2+Z5+anti-Vδ2). Then, each culture well was analyzed for the identification and measurement of spheroids as area **(A)** or volume **(B)**. The number of spheroids analyzed is indicated in the upper part of panel **(A)**; data are from experiments performed using six different Vδ2 T cell populations in triplicate wells for each donor. Each symbol in the plot indicates a single tumor cell spheroid. Bar: mean ± SEM of each group of measures. *p < 0.01; ***p < 0.0001; ns, not significant.

It is known that the recognition of IPP produced as a consequence of Zol effect on target cell metabolism is TCR dependent (4, 6–8). Thus, we assessed whether the anti-Vδ2 TCR-specific mAb BB3 can impair this recognition. As shown in Figure 6, the area (Figure 6A) and volume (Figure 6B) of HCT15 spheroids was markedly reduced when Zol and Vδ2 T cell populations were added to cell cultures. The addition of the anti-Vδ2-specific mAb significantly inhibited the reduction of spheroid size found in the presence of Vδ2 T cells and Zol (Figures 6A,B). This indicates that the 3D spheroid culture system allows the evaluation of the Zol-triggered Vδ2 T cell anti-tumor effect and of TCR involvement.

**Figure 7:**
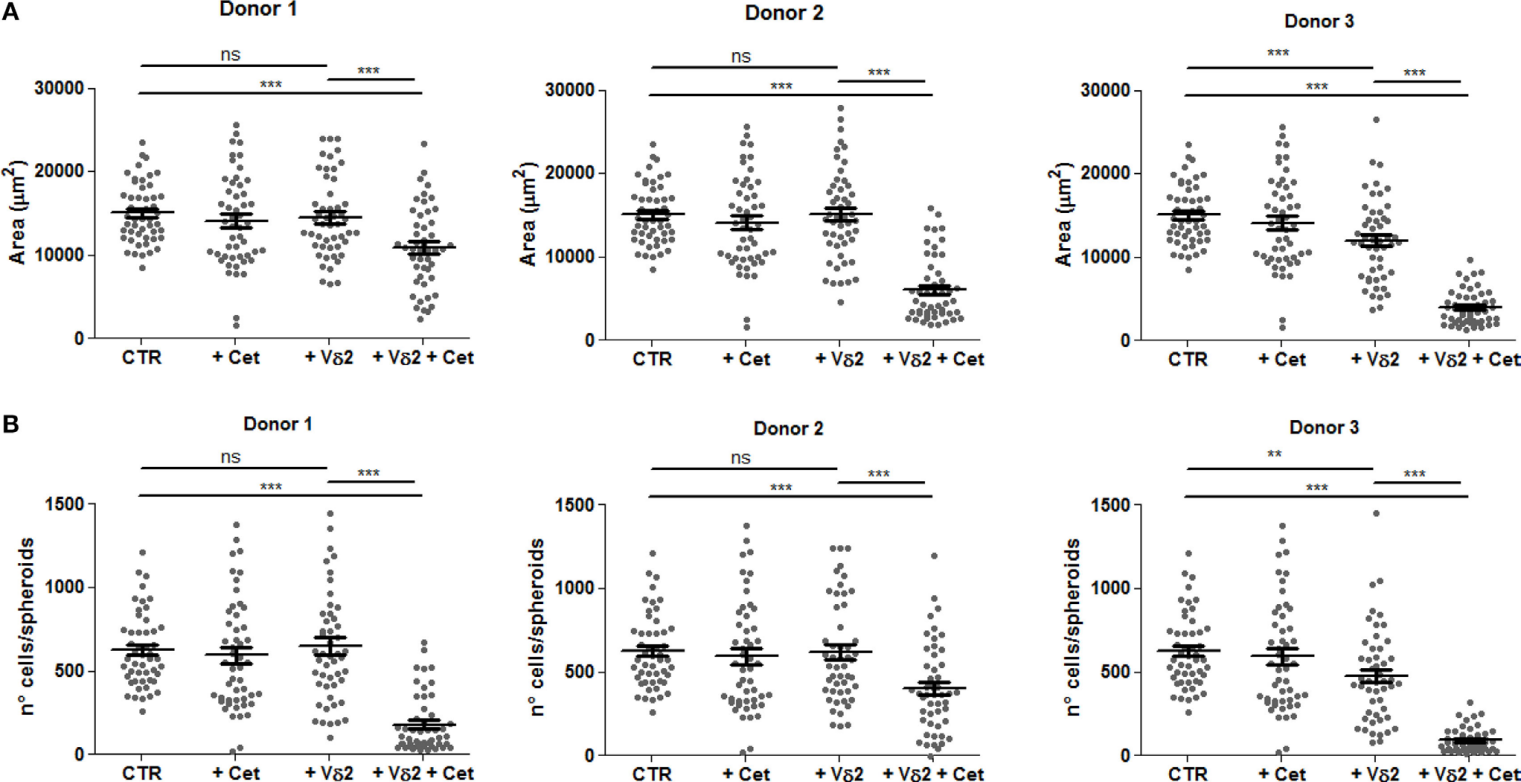
Vδ2 T cells can be triggered by the anti-epidermal growth factor receptor (EGFR) therapeutic antibody cetuximab to reduce CRC spheroids. HCT15 spheroids were generated in serum free medium supplemented with epithelial growth factor (10 ng/ml). On day 6, cultures were incubated for additional 24 h in medium without (CTR) or with the anti-EGFR antibody cetuximab (2 µg/ml, +Cet) or Vδ2 T cells (+Vδ2) or Vδ2 T cells and cetuximab (2 µg/ml, +Vδ2+Cet). Then, each culture well was analyzed for the identification and measurement of spheroids. Data are representative of experiments obtained with three different Vδ2 T cell populations in triplicate wells for each donor. Each symbol in the plot indicates a single tumor cell spheroid. Data are expressed as area of spheroids [**(A)**; μm^2^] or cell number in each spheroid **(B)**. Bar: mean ± SEM of that group of measures. **p < 0.01; ***p < 0.001; ns, not significant.

Vδ2 T cells can also be elicited to develop anti-tumor activity through ADCC; this can be obtained using therapeutic antibodies, such as the anti-EGFR huAb Cet (32, 33). Thus, we assessed whether the activation of ADCC triggered by Cet can reduce the size of tumor spheroids of HCT15 CRC. Vδ2 T cells were incubated with HCT15 spheroids and Cet added at the onset of the period of incubation; the size of spheroids was analyzed after 24 h. We found that when Cet and Vδ2 T cell populations were co-incubated with tumor spheroids, a strong reduction of spheroid size (Figure 7A) and cell number/spheroid (Figure 7B) was detected in experiments performed with Vδ2 T lymphocytes, enriched in CD16^+^ cells (Figure S1 in Supplementary Material, right histogram), from three different donors. Cet slightly reduced the spheroid size when used alone. These data suggest that Cet can trigger Vδ2 T lymphocytes to exert anti-CRC ADCC also in this 3D culture system.

## Discussion

In this paper, we analyzed the anti-tumor effect of Vδ2 T cells on different CRC cell lines organized in spheroid 3D structures. We show that 1. Spheroids can be a feasible and reliable system that allows the study of tumor cell interaction with effector lymphocytes in 3D cultures. 2. Zoledronate and cetuximab trigger Vδ2 T cells to kill and degrade spheroids of CRC cells. 3. The effect of the drug and of effector Vδ2 T cells can be evaluated and measured by computerized imaging.

The success rate of many new anti-cancer drugs and immunotherapies in clinical trials is surprisingly low, despite the promising effects obtained in preclinical studies (34). This might be due to the fact that 2D cultures poorly reflect the real tumor microenvironment, while in animals metabolism is not necessarily comparable to humans. This is particularly true in the case of CRC, where an appropriate animal model is lacking. The spatial organization, with different mechano-structural and physico-chemical features, has been proposed as the main factor responsible for the failure of conventional 2D culture systems (35, 36). Moreover, there is increasing evidence that human tumor development is not always reproducible and predictable in other animals, in particular when extremely artificial models are used, including humanized mice, needed to test anti-cancer immune response (18–20).

Among the 3D culture systems validated by EURL ECVAM as preclinical models, we used the simplest one, that is tumor cell spheroids (21–24, 37). Although tissue architecture is not entirely reproduced, the system is reminiscent of the small tumor cell clusters that may appear in the first stages of cancer development. Our present data indicate that we can count and measure the size of a large number of spheroids in replicate culture wells and different experimental conditions. Many CRC cell lines can be used in this assay (HCT15, SW480, HT29, Caco2, DLD1, HCT116, WiDr, SW620, LoVo, Colo205, Colo741, and T84), with the exception of some (SW48, Colo320DMF, and LS180) that may raise some difficulties in size measurement due to incomplete spheroid formation. Caco2 cells are even able to generate crypts-like structures that are more representative of CRC tissue. Acquisition of images, reconstruction of the whole culture well, computerized analysis of single spheroids and their measurement are easily performed with the CellSens computer system and manual definition of the ROI was reproducible without significant differences among operators; nevertheless, we are working on the construction of the macro of the ImageJ open source image processing program suitable for automated ROI definition, to achieve more precise, reliable, and quick sample analysis. Automated image analyses would also allow the evaluation of multiple *z*-planes and 3D reconstruction of spheroids. Nevertheless, in our system spheroid volume is calculated by the software starting from the maximal radius of the ROIs selected during the calibration procedure, thus giving a bona-fide 3D measure. Perimeter and area definition are already automatically determined by a measure tool of the CellSens software. Also, it is possible to calculate mathematically the number of cells in a given tumor spheroid, providing further information on tumor growth. Image analysis permits to evaluate the effects exerted by different EGFR ligands on CRC spheroid size, showing that EGF and TGFα are very efficient factors in increasing spheroid volume. Moreover, CRC spheroid size is related to ATP cell content and a direct relationship between ATP content and cell number can be defined, as parameter for cell metabolism and proliferation. Under these experimental conditions, several drugs, including Zol, can be tested for their anti-tumor efficiency: moreover, the system allows to distinguish among the response elicited in different donors as well.

Of note, we could analyze Vδ2 effector T lymphocyte activity on CRC cell spheroids. Some effects of anti-cancer lymphocytes on the killing of CRC cells isolated from tumor spheroids has been previously shown (31). However, in that report spheroids were first disaggregated and single cell suspensions of colon stem cells used in conventional cytolytic experiments. In our hands, under these experimental conditions, in some CRC cell lines a high percentage of cells show altered membrane permeability and this would advise against their use as target cells. Indeed, the large majority of assays designed to evaluate cytotoxicity are based on the different ability of cells with altered membrane permeability and cells with intact membrane to release an intracellular probe. Conversely, intact CRC spheroids displayed a membrane permeability superimposable to that of the original adherent cell line and are suitable for cytolytic assays. Moreover, crystal violet staining was used to check the viability of CRC cells of the whole spheroid and ATP measurement to evaluate cell metabolism and proliferation.

In this setting, a strong and reproducible reduction of spheroid size in all CRC cell lines was observed using Vδ2 T cells and Zol. The pharmacologic effect of Zol alone in reducing spheroid size was detected on HCT15, SW620, and DLD1 cell lines, while the reductive effect due to Vδ2 T cells, in the absence of Zol, was evident only using HCT15 as target cells. In any case, this 3D spheroid culture system allows the evaluation of drug and/or Vδ2 T cell anti-tumor effects; also, the system reveals any difference in the sensitivity of CRC cell types to drug or lymphocyte effects, and in the efficiency of anti-tumor effectors from distinct donors. Data from crystal violet staining were in line with image analysis of spheroid size; at variance, ATP measurement was reliable and reproducible only in CRC spheroids without Vδ2 T lymphocytes, conceivably due to the fact that in co-cultures the ATP of both cell types is measured, impairing the distinction between the fate of effectors and targets. Thus, we would advise crystal violet assay to check and support image analysis of tumor spheroid size to detect tumor killing exerted by Vδ2 T cells, alone or in combination of drugs, including Zol, at variance with ATP content that is useful only to test drug effects.

Using a specific mAb in inhibition experiments, we also show that Vδ2 TCR is involved in the spheroid reduction and killing elicited by Zol. Another important application of this 3D culture system is the evaluation of anti-CRC ADCC exerted by Vδ2 T lymphocytes triggered by the therapeutic anti-EGFR huAb cetuximab (Cet). Indeed, when Cet and Vδ2 T cell populations were co-incubated with CRC spheroids a strong reduction of their size and cell number/spheroid was detected in experiments performed with Vδ2 T lymphocytes, enriched in CD16^+^ cells. Cet slightly reduced the spheroid size when used alone. This suggests that also therapeutic mAbs other than Cet can be evaluated for their efficacy in producing a direct anti-tumor cytotoxic effect or ADCC in this 3D culture system. A limitation of this model is represented by the absence of stromal or myeloid or other types of neighboring cells that compose the tumor microenvironment; however, the system potentially allows the setting of mixed spheroids, e.g., using a core of stromal cells and an envelope of cancer cells. Mixed spheroids with different degree of complexity, made of CRC cell lines and cancer-associated fibroblasts (CAF), with or without PBMC, have been reported (38). In this mixed spheroid system, CAF conferred to co-cultured CRC cells reduced chemosensitivity, supporting that tumor microenvironment deeply influences the outcome of drug treatments. Also, it is known that stromal cells can down-regulate the function of immune effector cells, although we have reported that zoledronate can prevent this inhibition, allowing the occurrence of effector lymphocyte cell function (1, 29).

In conclusion, mixed lymphocyte-CRC spheroids represent a reliable, reproducible, and cheap 3D culture system that allows the evaluation and measurement of anti-cancer drugs that exploit the immune response. Image computerized analysis guarantees the precise and unbiased analysis of data, although some efforts are still needed to reach a good automated ROI definition and image assessment of the whole spheroid.

## Ethics Statement

Peripheral blood mononuclear cells (PBMC) were obtained from healthy adult donor’s buffy coat upon institutional informed consent signed at the time of donation and EC approval PR163REG2014 of the Regional Ethic Committee.

## Author Contributions

SV carried out cell isolation, generation, and culture of spheroids, and functional assays; SV, MZ, and AP performed immunofluorescence and image analyses, designed the work, and wrote the paper. AP takes primary responsibility for the paper content.

## Conflict of Interest Statement

The authors declare that the research was conducted in the absence of any commercial or financial relationships that could be construed as a potential conflict of interest.

## Supplementary Material

Figure S1 Tumor cell spheroids formation and three-dimensional (3D) co-culture system with Vδ2 T cells. **(A)** Schematic representation and time schedule of the 3D culture system. CRC cell lines were cultured in serum free medium supplemented with 10 ng/ml of epithelial growth factor in very low-adherent 96-well flat-bottomed microplates (10^4^ cells/well). Cell cultures were checked every day (a) by inverted IX70 microscope. On day 6 (b) either Zol (5 µM) or Vδ2 T cells or Zol and Vδ2 T cells were added and the spheroids analyzed after additional 24 h of incubation (c). Then, image analysis, ATP content measurement, and crystal violet assay were performed. **(B)** Bright field microscopic images of the cultures described in panel **(A)**. (a) CRC spheroid. (b) Vδ2 T cells added to CRC spheroids. (c) Zol and Vδ2 T cells added to CRC spheroids. **(C)** Phenotype of the Vδ2 T cell populations obtained culturing peripheral blood mononuclear cell with Zol and then used in the assay described in panel **(A)**. Left dot plot: double staining and FACS analysis of Vδ2 T cells with anti-CD3 and anti-Vδ2 mAbs at 21 days of culture; in each quadrant, percentage of cells. Central graph: percentages of Vδ2 T cells at the indicated time points; each dot represents a single donor. Bars: mean ± SD. Right graph: expression of CD16 antigen on Vδ2 T cells (day 21) assessed by immunofluorescence and FACS analysis. Dark grey: negative control with unrekated APC-Ig. Log red fluorescence intensity (a.u.) vs cell number. The percentage of positive cells is indicated. One representative donor out of six.

Figure S2 Phenotype of CRC cell lines and CRC spheroids. **(A)** Immunofluorescence performed on the indicated CRC cell lines with the anti-CD133-specific monoclonal antibody (mAb) followed by Alexafluor647 GAM isotype specific antiserum. Dark gray histogram in each panel: fluorescence of cells stained with the second reagent alone. Light gray histogram: fluorescence of cells stained with anti-CD133 mAb. **(B)** Immunofluorescence performed on SW480 cell line cultured under conventional conditions (upper row) or as spheroids (lower row), with the anti-ICAM1 mAb, followed by Alexafluor647 GAM isotype specific antiserum, or the Fc-NKG2D or the Fc-DNAM1 chimeras, followed by Alexafluor647 anti-human specific antiserum. Data are expressed as log far red MFI in arbitrary units (a.u.).

Figure S3 Measurement of perimeter and area of CRC spheroids by different operators. **(A,B)** The perimeter **(A)** and area **(B)** of SW620 (left measures in each panel) or HCT15 (right measures in each panel) spheroids were analyzed independently by three operators (OP1, OP2, and OP3), calculated as in Figure 2 and data plotted with Graph Pad PRISM software. Each symbol indicates a region of interest (ROI) which in turn corresponds to a single spheroid. Bar in each plot shows the mean ± SD of that group of measures.

Figure S4 ATP content and propidium iodide (PI) staining in CRC spheroids. **(A)** ATP content, expressed as µM calculated referring to luminescence of a standard curve, in the CRC cell lines HCT15, HT29, Caco2, and SW480, seeded at the indicated number of cells/well. Data are the mean of 6-well replicates for each culture condition. **(B)** PI staining of HCT15 (upper row), SW620 (central row), and SW480 (lower row) CRC cell lines. Left histograms: adherent cells in conventional cultures; middle histograms: disaggregated spheroids; and right histograms: spheroids recovered and cultured in adherent plates. The percentages of PI positive cells are indicated in each histogram.

Figure S5 Effect of Vδ2 T cell populations from different donors on spheroid size. HCT15 spheroids were obtained after culture for 6 days in serum free medium supplemented with epithelial growth factor (10 ng/ml). Cultures were incubated for additional 24 h in medium without (CTR) or with 5 µM Zol (+Z5) or Vδ2 T cells (+Vδ2) or Vδ2 T cells + 5μM Zol (+Vδ2+Z5). Then, each culture well was analyzed for the identification and measurement of spheroids. Data are from experiments performed using six different Vδ2 T cell populations (donors 1, 2, 3, 4, 5, and 6) in triplicate wells for each donor and are expressed in µm^2^. Each symbol in the plot indicates a single tumor cell spheroid. Bars: mean ± SEM. **p* < 0.01; ***p* < 0.001, ****p* < 0.0001; ns, not significant.

## References

[1] Poggi A Musso A Dapino I Zocchi MR. Mechanisms of tumor escape from immune system: role of mesenchymal stromal cells. Immunol Lett (2014) 159:55–72. 10.1016/j.imlet.2014.03.001 24657523

[2] Marin-Acevedo JA Soyano AE Dholaria B Knutson KL Lou Y Cancer immunotherapy beyond immune checkpoint inhibitors. J Hematol Oncol (2018) 11:8 10.1186/s13045-017-0552-6 29329556 PMC5767051

[3] Engelhard VH Rodriguez AB Mauldin IS Woods AN Peske JD Slingluff CL Jr. Immune cell infiltration and tertiary lymphoid structures as determinants of antitumor immunity. J Immunol (2018) 200:432–42. 10.4049/jimmunol.1701269 29311385 PMC5777336

[4] Vantourout P Hayday A. Six-of-the-best: unique contributions of γδ T cells to immunology. Nat Rev Immunol (2013) 13:88–100. 10.1038/nri3384 23348415 PMC3951794

[5] Lo Presti E Pizzolato G Gulotta E Cocorullo G Gulotta G Dieli F Current advances in γδ T cell-based tumor immunotherapy. Front Immunol (2017) 8:1401 10.3389/fimmu.2017.01401 29163482 PMC5663908

[6] Vavassori S Kumar A Wan GS Ramanjaneyulu GS Cavallari M El Daker S Butyrophilin 3A1 binds phosphorylated antigens and stimulates human γδ T cells. Nat Immunol (2013) 14:908–16. 10.1038/ni.2665 23872678

[7] Allison TJ Winter CC Fournié JJ Bonneville M Garboczi DN. Structure of a human gammadelta T-cell antigen receptor. Nature (2001) 411:820–4. 10.1038/35081115 11459064

[8] Gober HJ Kistowska M Angman L Jenö P Mori L De Libero G. Human T cell receptor gammadelta cells recognize endogenous mevalonate metabolites in tumor cells. J Exp Med (2003) 197:163–8. 10.1084/jem.20021500 12538656 PMC2193814

[9] Morita CT Jin C Sarikonda G Wang H. Nonpeptide antigens, presentation mechanisms, and immunological memory of human Vgamma2Vdelta2 T cells: discriminating friend from foe through the recognition of prenyl pyrophosphate antigens. Immunol Rev (2007) 215:59–76. 10.1111/j.1600-065X.2006.00479.x 17291279

[10] Harly C Peigné CM Scotet E Molecules and mechanisms implicated in the peculiar antigenic activation process of human Vg9Vd2 T cells. Front Immunol (2015) 5:657 10.3389/fimmu.2014.00657 25601861 PMC4283718

[11] Kondo M Izumi T Fujieda N Kondo A Morishita T Matsushita H Expansion of human peripheral blood γδ T cells using zoledronate. J Vis Exp (2011) 55:e3182 10.3791/3182 PMC3230197 21931292

[12] Das H Wang L Kamath A Bukowski JF. Vgamma2Vdelta2 T-cell receptor-mediated recognition of aminobisphosphonates. Blood (2001) 98:1616–8. 10.1182/blood.V98.5.1616 11520816

[13] Byun JH Jang S Lee S Park S Yoon HK Yoon BH The efficacy of bisphosphonates for prevention of osteoporotic fracture: an update meta-analysis. J Bone Metab (2017) 24:37–49. 10.11005/jbm.2017.24.1.37 28326300 PMC5357611

[14] Heymann D Ory B Gouin F Green JR Rédini F. Bisphosphonates: new therapeutic agents for the treatment of bone tumors. Trends Mol Med (2004) 10:337–43. 10.1016/j.molmed.2004.05.007 15242682

[15] Thompson K Roelofs AJ Jauhiainen M Mönkkönen H Mönkkönen J Rogers MJ Activation of γδ T cells by bisphosphonates. Adv Exp Med Biol (2010) 658:11–20. 10.1007/978-1-4419-1050-9_2 19950011

[16] Lafont V Liautard J Liautard JP Favero J. Production of TNF-alpha by human V gamma 9V delta 2 T cells via engagement of Fc gamma RIIIA, the low affinity type 3 receptor for the Fc portion of IgG, expressed upon TCR activation by nonpeptidic antigen. J Immunol (2001) 166:7190–9. 10.4049/jimmunol.166.12.7190 11390467

[17] Zhao Y Niu C Cui J. Gamma-delta (γδ) T cells: friend or foe in cancer development? J Transl Med (2018) 16:3. 10.1186/s12967-017-1378-2 29316940 PMC5761189

[18] Landgraf M McGovern JA Friedl P Hutmacher DW. Rational design of mouse models for cancer research. Trends Biotechnol (2018) 36:242–51. 10.1016/j.tibtech.2017.12.001 29310843

[19] Westbrook AM Szakmary A Schiestl RH. Mouse models of intestinal inflammation and cancer. Arch Toxicol (2016) 90:2109–30. 10.1007/s00204-016-1747-2 27311821

[20] Snider AJ Bialkowska AB Ghaleb AM Yang VW Obeid LM Hannun YA. Murine model for colitis-associated cancer of the colon. Methods Mol Biol (2016) 1438:245–54. 10.1007/978-1-4939-3661-8_14 27150094 PMC5657253

[21] Akhtar A. The flaws and human harms of animal experimentation. Camb Q Healthc Ethics (2015) 24:407–19. 10.1017/S0963180115000079 26364776 PMC4594046

[22] Horstmann E McCabe MS Grochow L Yamamoto S Rubinstein L Budd T Risks and benefits of phase 1 oncology trials, 1991 through 2002. N Engl J Med (2005) 352:895–904. 10.1056/NEJMsa042220 15745980

[23] Ellis LM Fidler IJ Finding the tumor copycat. Therapy fails, patients don’t. Nat Med (2010) 16:974–5. 10.1038/nm0910-974 20823880

[24] Enna SJ Williams M. Defining the role of pharmacology in the emerging world of translational research. Adv Pharmacol (2009) 57:1–30. 10.1016/S1054-3589(08)57001-3 20230758

[25] Verjans ET Doijen J Luyten W Landuyt B Schoofs L. Three-dimensional cell culture models for anticancer drug screening: worth the effort? J Cell Physiol (2018) 233:2993–3003. 10.1002/jcp.26052 28618001

[26] Sant S Johnston PA. The production of 3D tumor spheroids for cancer drug discovery. Drug Discov Today Technol (2017) 23:27–36. 10.1016/j.ddtec.2017.03.002 28647083 PMC5497458

[27] Rodrigues T Kundu B Silva-Correia J Kundu SC Oliveira JM Reis RL Emerging tumor spheroids technologies for 3D in vitro cancer modeling. Pharmacol Ther (2018) 184:201–11. 10.1016/j.pharmthera.2017.10.018 29097309

[28] Zanoni M Piccinini F Arienti C Zamagni A Santi S Polico R 3D tumor spheroid models for in vitro therapeutic screening: a systematic approach to enhance the biological relevance of data obtained. Sci Rep (2016) 6:19103. 10.1038/srep19103 26752500 PMC4707510

[29] Zocchi MR Costa D Venè R Tosetti F Ferrari N Minghelli S Zoledronate can induce colorectal cancer microenvironment expressing BTN3A1 to stimulate effector γδ T cells with antitumor activity. Oncoimmunology (2017) 6:e1278099. 10.1080/2162402X.2016.1278099 28405500 PMC5384426

[30] Ciccone E Ferrini S Bottino C Viale O Prigione I Pantaleo G A monoclonal antibody specific for a common determinant of the human T cell receptor gamma/delta directly activates CD3+WT31- lymphocytes to express their functional program(s). J Exp Med (1988) 168:1–11. 10.1084/jem.168.1.1 2456364 PMC2188975

[31] Todaro M D’Asaro M Caccamo N Iovino F Francipane MG Meraviglia S Efficient killing of human colon cancer stem cells by gammadelta T lymphocytes. J Immunol (2009) 182:7287–96. 10.4049/jimmunol.0804288 19454726

[32] Matić IZ Kolundžija B Damjanović A Spasić J Radosavljević D Đorđić Crnogorac M Peripheral white blood cell subsets in metastatic colorectal cancer patients treated with cetuximab: the potential clinical relevance. Front Immunol (2018) 8:1886. 10.3389/fimmu.2017.01886 29354119 PMC5758541

[33] Turin I Delfanti S Ferulli F Brugnatelli S Tanzi M Maestri M In vitro killing of colorectal carcinoma cells by autologous activated NK cells is boosted by anti-epidermal growth factor receptor-induced ADCC regardless of RAS mutation status. J Immunother (2018) 41:190–200. 10.1097/CJI.0000000000000205 29293164

[34] Arrowsmith J Trial watch: phase II failures: 2008-2010. Nat Rev Drug Discov (2011) 10:328–9. 10.1038/nrd3439 21532551

[35] Fuster MM Esko JD. The sweet and sour of cancer: glycans as novel therapeutic targets. Nat Rev Cancer (2005) 5:526–42. 10.1038/nrc1649 16069816

[36] Tung YC Hsiao AY Allen SG Torisawa YS Ho M Takayama S. High-throughput 3D spheroid culture and drug testing using a 384 hanging drop array. Analyst (2011) 136:473–8. 10.1039/c0an00609b 20967331 PMC7454010

[37] Shanks N Greek R Greek J. Are animal models predictive for humans? Philos Ethics Humanit Med (2009) 4:2. 10.1186/1747-5341-4-2 19146696 PMC2642860

[38] Hoffmann OI Ilmberger C Magosch S Joka M Jauch KW Mayer B. Impact of the spheroid model complexity on drug response. J Biotechnol (2015) 205:14–23. 10.1016/j.jbiotec.2015.02.029 25746901

