# Corrigendum: Zoledronate Triggers Vδ2 T Cells to Destroy and Kill Spheroids of Colon Carcinoma: Quantitative Image Analysis of Three-Dimensional Cultures

**DOI:** 10.3389/fimmu.2018.01343

**Published:** 2018-06-13

**Authors:** Serena Varesano, Maria Raffaella Zocchi, Alessandro Poggi

**Affiliations:** ^1^Molecular Oncology and Angiogenesis Unit, Ospedale Policlinico San Martino, Genoa, Italy; ^2^Division of Immunology, Transplants and Infectious Diseases, San Raffaele Scientific Institute, Milan, Italy

**Keywords:** CRC, spheroid, γδ T cells, epidermal growth factor receptor, antibody-dependent cellular cytotoxicity, zoledronate

There is an error in the Funding statement. The correct name for one of the funders is Ministero della Salute. The correct Funding statement is: This work has been partially supported by grants from AIRC to AP (IG15483) and AIRC to MZ (IG17074) and from 5xmille 2012 Ministero della Salute, 5xmille 2013 Ministero della Salute, 5xmille 2014 Ministero della Salute, and 5xmille 2015 Ministero della Salute to AP. The authors apologize for this error and state that this does not change the scientific conclusions of the article in any way.

The original article has been updated.

## Conflict of Interest Statement

The authors declare that the research was conducted in the absence of any commercial or financial relationships that could be construed as a potential conflict of interest.

