# Supplementary figures and images for: Zoledronate Triggers Vδ2 T Cells to Destroy and Kill Spheroids of Colon Carcinoma: Quantitative Image Analysis of Three-Dimensional Cultures
Source: Front Immunol. 2018 May 8;9:998. doi: 10.3389/fimmu.2018.00998 (PMC5951939; doi:10.3389/fimmu.2018.00998)

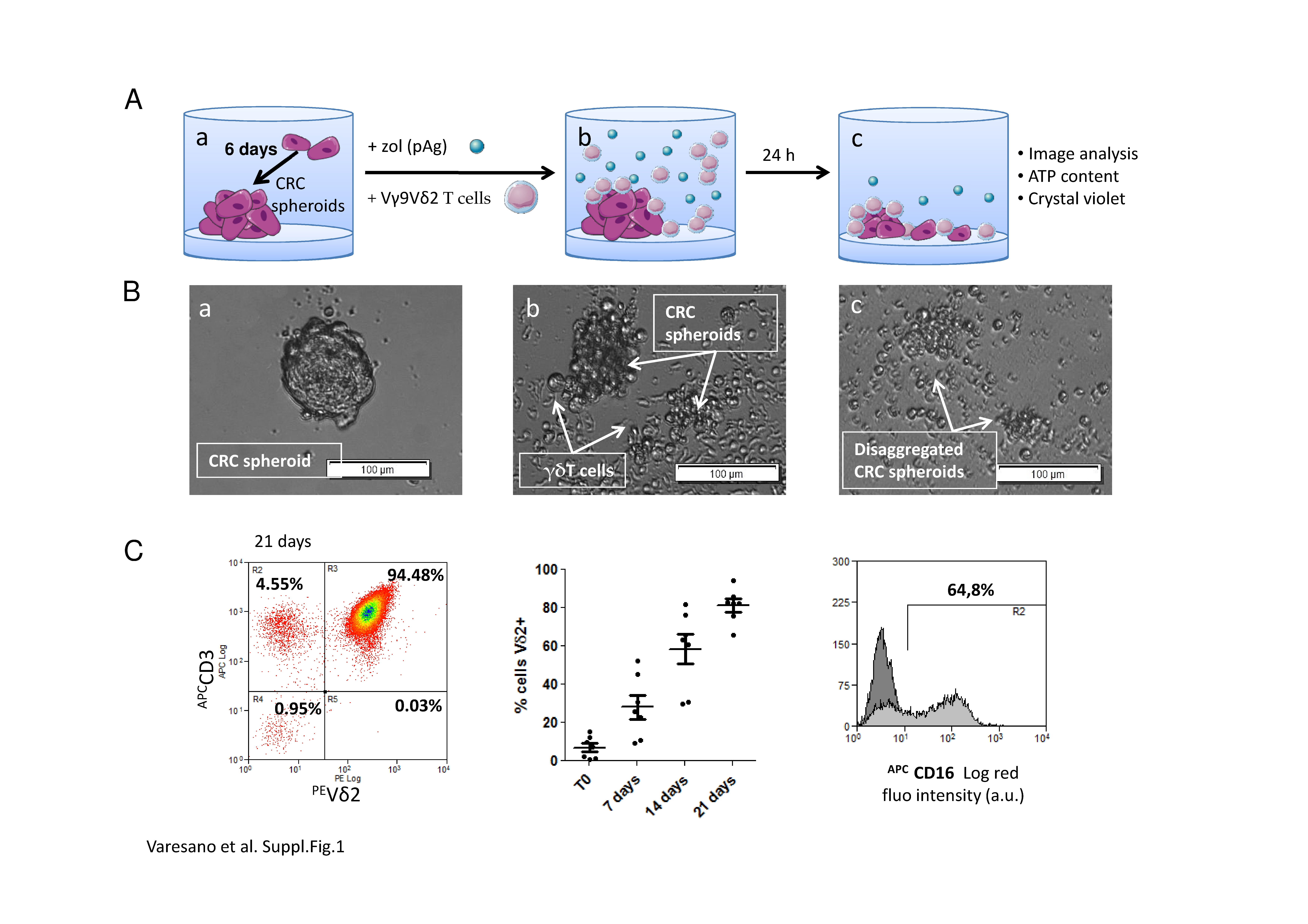

Supplement: Figure S1 — Tumor cell spheroids formation and three-dimensional (3D) co-culture system with Vδ2 T cells. (A) Schematic representation and time schedule of the 3D culture system. CRC cell lines were cultured in serum free medium supplemented with 10 ng/ml of epithelial growth factor in very low-adherent 96-well flat-bottomed microplates (104 cells/well). Cell cultures were checked every day (a) by inverted IX70 microscope. On day 6 (b) either Zol (5 µM) or Vδ2 T cells or Zol and Vδ2 T cells were added and the spheroids analyzed after additional 24 h of incubation (c). Then, image analysis, ATP content measurement, and crystal violet assay were performed. (B) Bright field microscopic images of the cultures described in panel (A). (a) CRC spheroid. (b) Vδ2 T cells added to CRC spheroids. (c) Zol and Vδ2 T cells added to CRC spheroids. (C) Phenotype of the Vδ2 T cell populations obtained culturing peripheral blood mononuclear cell with Zol and then used in the assay described in panel (A). Left dot plot: double staining and FACS analysis of Vδ2 T cells with anti-CD3 and anti-Vδ2 mAbs at 21 days of culture; in each quadrant, percentage of cells. Central graph: percentages of Vδ2 T cells at the indicated time points; each dot represents a single donor. Bars: mean ± SD. Right graph: expression of CD16 antigen on Vδ2 T cells (day 21) assessed by immunofluorescence and FACS analysis. Dark grey: negative control with unrekated APC-Ig. Log red fluorescence intensity (a.u.) vs cell number. The percentage of positive cells is indicated. One representative donor out of six. [file image_1.jpeg]

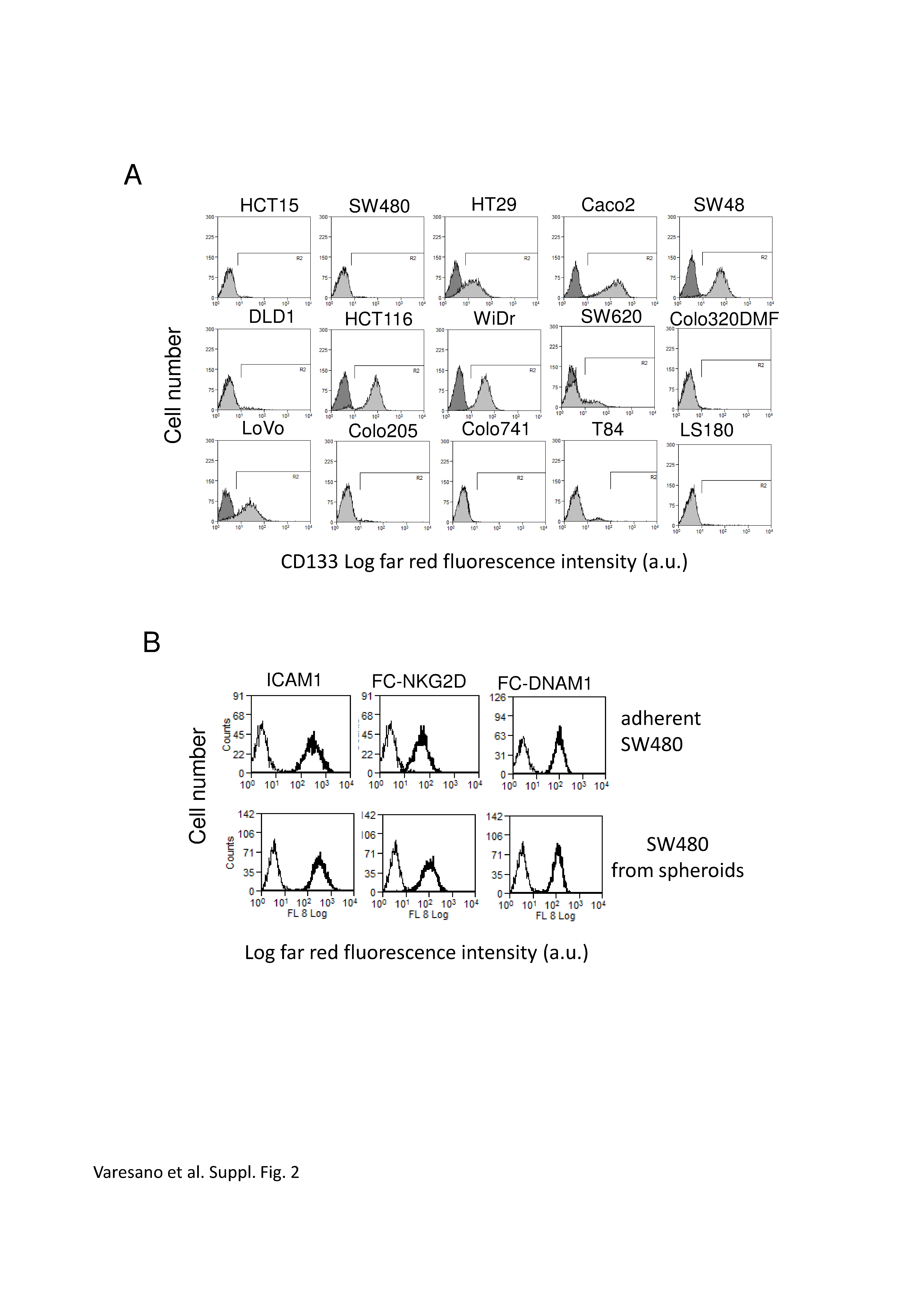

Supplement: Figure S2 — Phenotype of CRC cell lines and CRC spheroids. (A) Immunofluorescence performed on the indicated CRC cell lines with the anti-CD133-specific monoclonal antibody (mAb) followed by Alexafluor647 GAM isotype specific antiserum. Dark gray histogram in each panel: fluorescence of cells stained with the second reagent alone. Light gray histogram: fluorescence of cells stained with anti-CD133 mAb. (B) Immunofluorescence performed on SW480 cell line cultured under conventional conditions (upper row) or as spheroids (lower row), with the anti-ICAM1 mAb, followed by Alexafluor647 GAM isotype specific antiserum, or the Fc-NKG2D or the Fc-DNAM1 chimeras, followed by Alexafluor647 anti-human specific antiserum. Data are expressed as log far red MFI in arbitrary units (a.u.). [file image_2.jpeg]

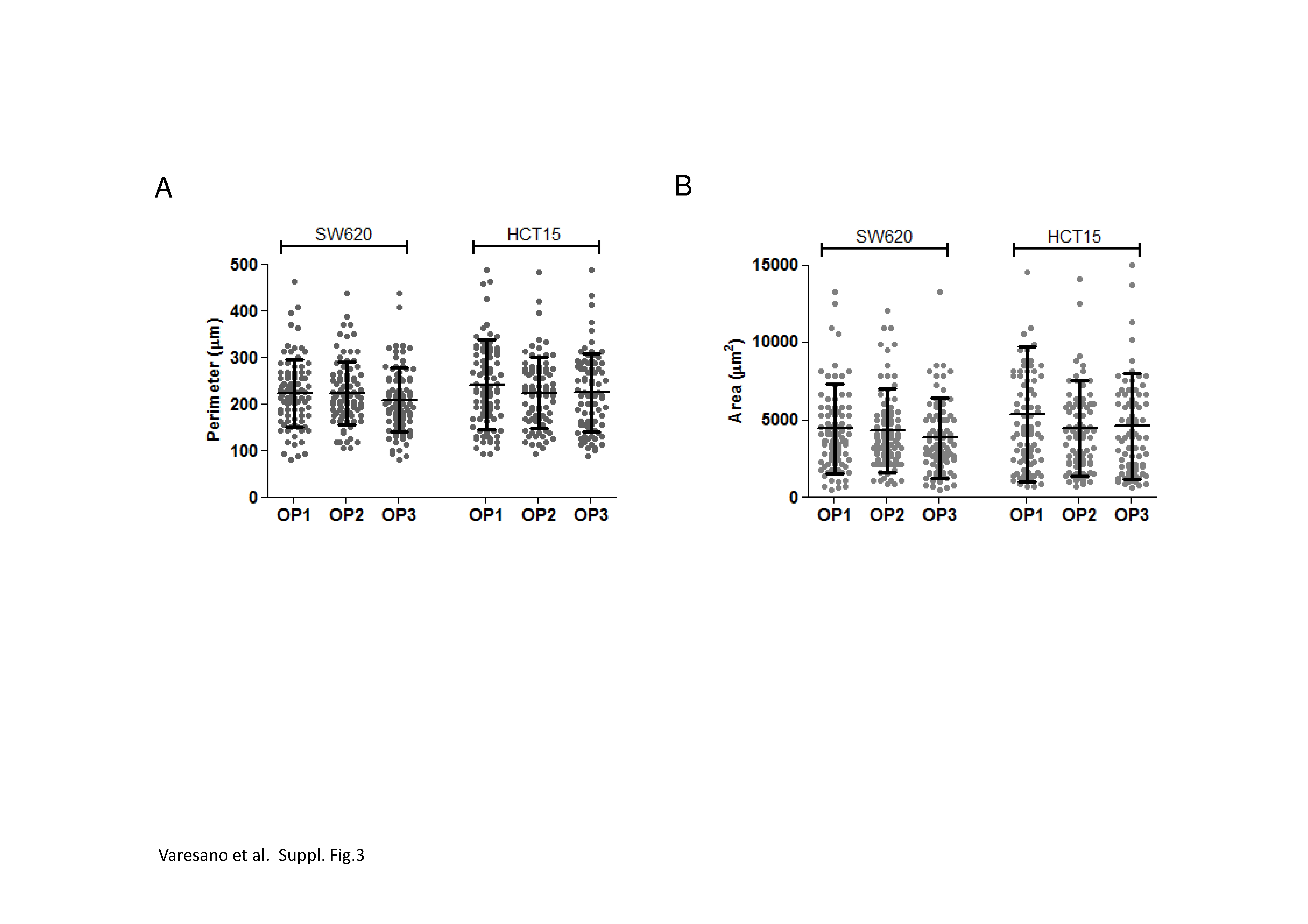

Supplement: Figure S3 — Measurement of perimeter and area of CRC spheroids by different operators. (A,B) The perimeter (A) and area (B) of SW620 (left measures in each panel) or HCT15 (right measures in each panel) spheroids were analyzed independently by three operators (OP1, OP2, and OP3), calculated as in Figure 2 and data plotted with Graph Pad PRISM software. Each symbol indicates a region of interest (ROI) which in turn corresponds to a single spheroid. Bar in each plot shows the mean ± SD of that group of measures. [file image_3.jpeg]

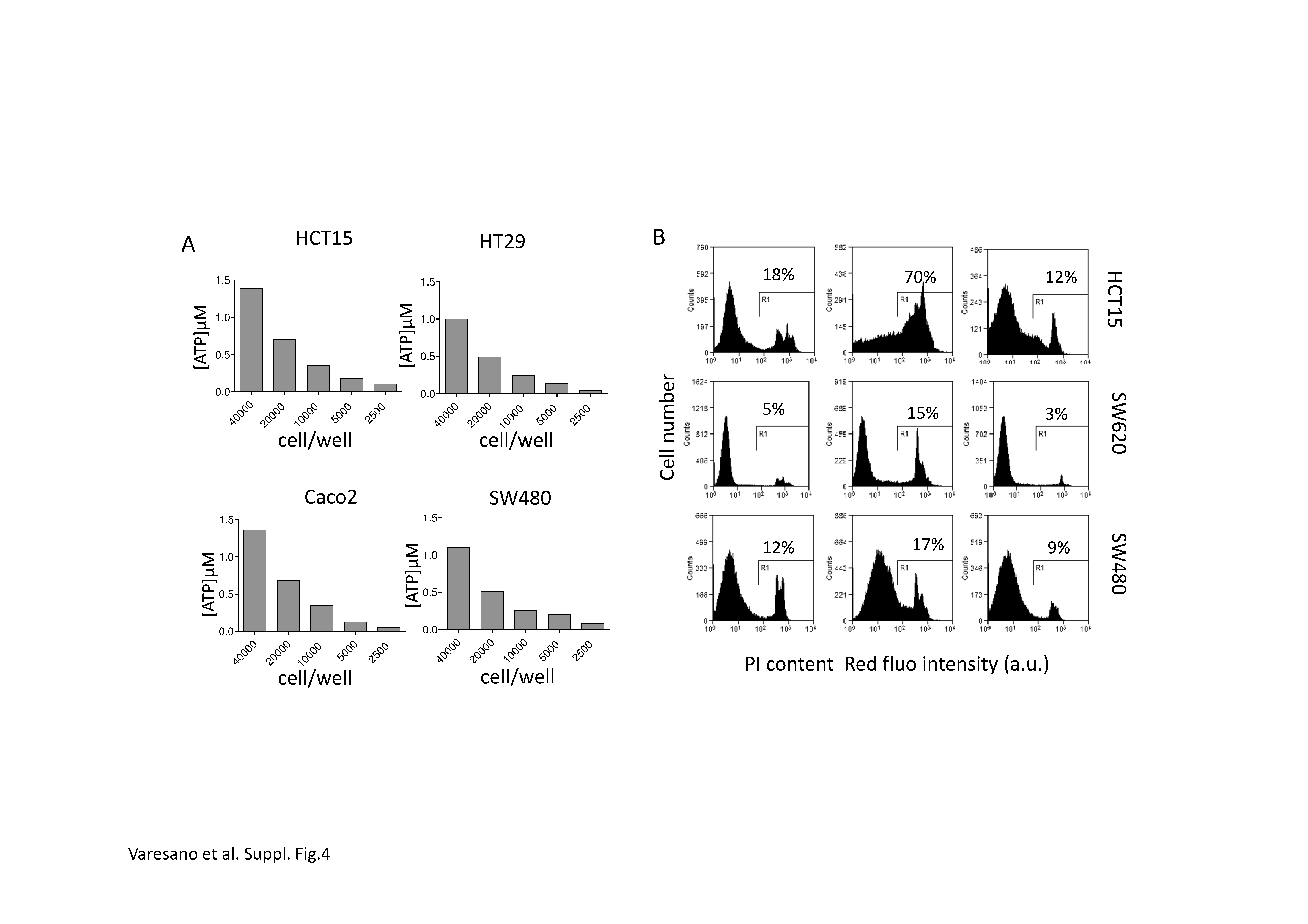

Supplement: Figure S4 — ATP content and propidium iodide (PI) staining in CRC spheroids. (A) ATP content, expressed as µM calculated referring to luminescence of a standard curve, in the CRC cell lines HCT15, HT29, Caco2, and SW480, seeded at the indicated number of cells/well. Data are the mean of 6-well replicates for each culture condition. (B) PI staining of HCT15 (upper row), SW620 (central row), and SW480 (lower row) CRC cell lines. Left histograms: adherent cells in conventional cultures; middle histograms: disaggregated spheroids; and right histograms: spheroids recovered and cultured in adherent plates. The percentages of PI positive cells are indicated in each histogram. [file image_4.jpeg]

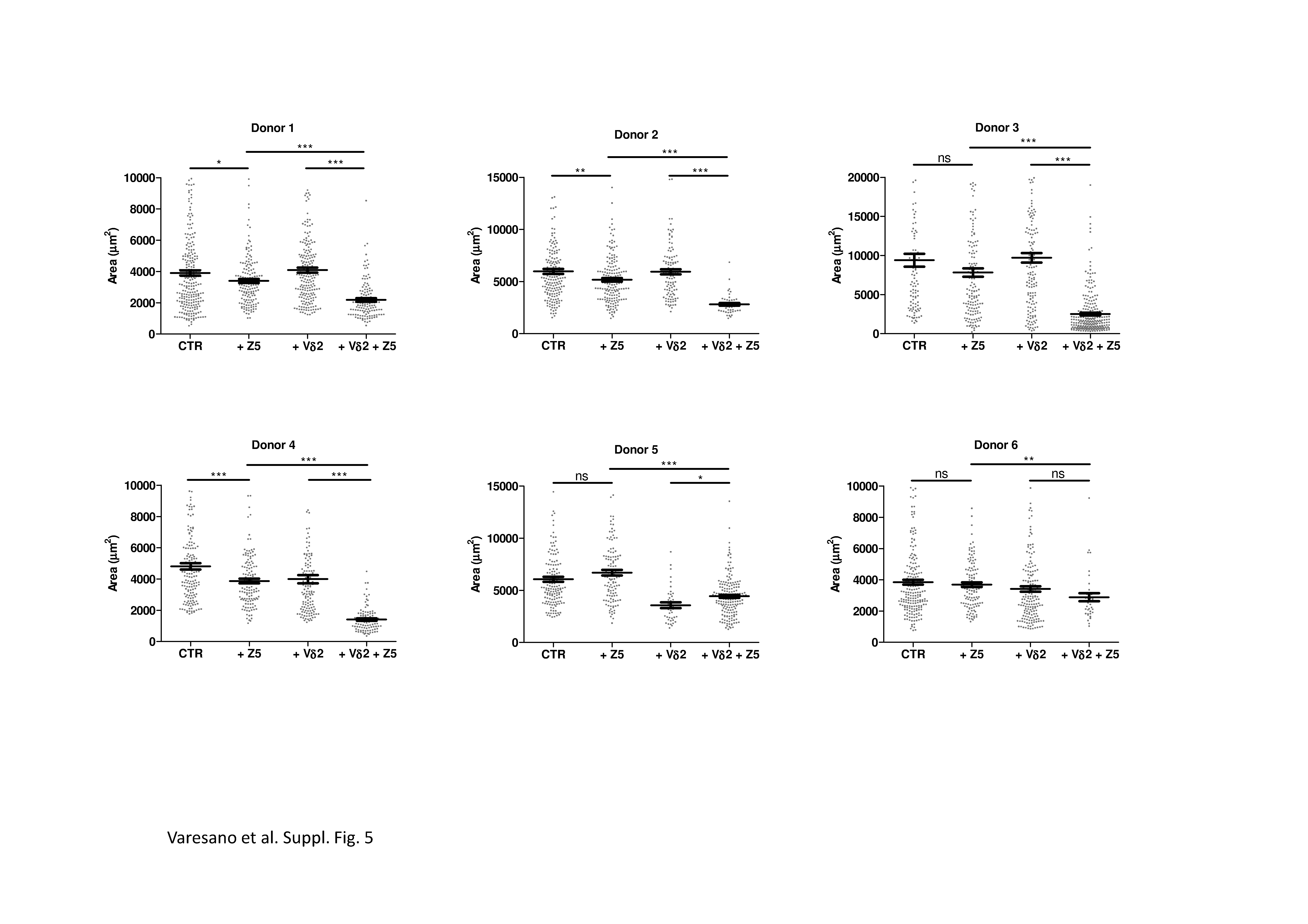

Supplement: Figure S5 — Effect of Vδ2 T cell populations from different donors on spheroid size. HCT15 spheroids were obtained after culture for 6 days in serum free medium supplemented with epithelial growth factor (10 ng/ml). Cultures were incubated for additional 24 h in medium without (CTR) or with 5 µM Zol (+Z5) or Vδ2 T cells (+Vδ2) or Vδ2 T cells + 5μM Zol (+Vδ2+Z5). Then, each culture well was analyzed for the identification and measurement of spheroids. Data are from experiments performed using six different Vδ2 T cell populations (donors 1, 2, 3, 4, 5, and 6) in triplicate wells for each donor and are expressed in µm2. Each symbol in the plot indicates a single tumor cell spheroid. Bars: mean ± SEM. *p < 0.01; **p < 0.001, ***p < 0.0001; ns, not significant. [file image_5.jpeg]
